# Effect of intake manifold geometry on cylinder-to-cylinder variation and tumble enhancement in gasoline direct injection engine

**DOI:** 10.1038/s41598-022-24079-8

**Published:** 2022-11-18

**Authors:** Jisoo Shin, Donghwan Kim, Yousang Son, Sungwook Park

**Affiliations:** 1 Department of Mechanical Convergence Engineering, Graduate School of Hanyang University, Seoul, 04763 Republic of Korea; 2 Hyundai Motors Group, Hwaseong, Gyeonggi-do 18280 Republic of Korea; 3 School of Mechanical Engineering, Hanyang University, 222 Wangsimni-ro, Seongdong-gu, Seoul, 04763 Republic of Korea

**Keywords:** Engineering, Mechanical engineering

## Abstract

In this study, the effect of intake manifold geometry on cylinder-to-cylinder variation was investigated considering the volumetric efficiency, early tumble development, turbulent kinetic energy, and spark plug gap velocity using computational fluid dynamic program, CONVERGE v2.4. The simulation model was validated based on the PIV experiment in the cylinder and Mie-scattering experiment of intake manifold, and its results agreed well with the experiment results. The curved intake manifold and straight manifold were compared. As a result, it was advantageous for cylinder-to-cylinder variation in the straight intake manifold compared to the curved intake manifold in perspective of volumetric efficiency which were a maximum deviation of 1.7% in curved manifold and 0.6% in straight manifold. And the straight manifold had an effect of the strengthening the in-cylinder flow, so that the turbulent kinetic energy near TDC was increased to maximum 11% than curved manifold. And considering the effect of manifold curve radius on in-cylinder flow intensity in straight manifold, with increasing engine speed, the in-cylinder flow intensified during compression with decreasing the intake manifold radius due to the short distance between manifold inlet and port. Especially at 2000 rpm, the tumble ratio increased 55% at intake manifold radius of 10 cm than of 7 cm at bTDC 280 deg. Therefore, for the purpose of enhancing the in-cylinder flow near spark plug timing, shortened distance between intake manifold inlet and port and increasing the manifold radius is required.

## Introduction

In current transportation market, the role of internal combustion engines is still important^1^ because the electric vehicles have problems to overcome, such as battery problem and power generation^2,3^. There is great scope for even further improvements in engines with advances in combustion technologies to meet the stringent environmental regulations^4,5^. Among internal combustion engines, gasoline direct injection (GDI) engines have the advantages of fuel economy, transient response, and precise air–fuel ratio control^6,7^. Thus, many studies have been focused on improving engine efficiency^8^ and reducing the exhaust emissions of GDI engines^9,10^.

It is known that the engine system needs to be optimally designed and to be adjusted adequate engine operating parameters to ensuring engine performance^11,12^. However, reducing the cylinder-to-cylinder variation should be basically secured to realize the optimized engine performance for a multi-cylinder engine.

The cylinder-to-cylinder variation is closely associated with the intake process in GDI engines, and it is inevitable, but it has a crucial impact on engine performance^13^. Additionally, in the GDI engine system, the tumble flow in the cylinder generated by intake process determine the turbulent intensity, mixing process, and further the combustion process^14^. Therefore, the intake system needs to be developed based on the difference of charged air mass and turbulent intensity by intake manifold geometry between cylinders causes the variation of combustion characteristics^15^.

The intake manifold geometry can be varied depending on the engine mounting space and the number of cylinders in the engine design, and it determines the intake manifold performance^16^. The in-cylinder flow characteristic is bound to be affected by the intake manifold geometry because the flow path is formed by the intake manifold inlet to the in-cylinder. Therefore, an appropriate intake manifold design that can minimize cylinder-to-cylinder variation and form an optimal flow that ensures high tumble intensity is required^17^. Some studies about intake manifold design have been conducted to enhance intake manifold performance.

Talati and Aliakbari et al.^18^ are investigated about the optimization of intake manifold runner length with varying engine speed. They suggested the variable-length intake manifold. And it was effective to improve the volumetric efficiency and thermal efficiency to 6.33% and 1.77% respectively within a certain operating range. Gocmen and Soyhen^19^ performed CFD simulation to evaluate the intake manifold geometry performance in a diesel engine with respect to reducing the pressure loss in intake manifold. The computational domain was the intake manifold region. They provide the new manifold design for reducing pressure loss of each cylinder in diesel engine.

 The intake manifold design for a GDI engine, however, needs to evaluate the aspect of not only volumetric efficiency and pressure drop but in-cylinder flow characteristics. The in-cylinder flow development is closely related to the mixture preparation process in GDI engine^20^. And Nashiyama and Furui^21^ found the importance of the spark plug gap velocity near spark timing for GDI engine combustion characteristics using visualization analysis. In the study of Benajes and Garcia et al.^22^, the spark plug gap velocity had a significant effect on determining the direction and speed of early flame propagation which is affect to the overall combustion phase. Raja and Selvam^23^ found that the intake manifold geometry subsequently affected flow, air–fuel mixing, and combustion. Considering the gasoline engine system characteristics, the intake manifold geometry performance should be analyzed comprehensively taking into account in-cylinder flow and spark plug gap flow. And the cylinder-to-cylinder variation for different intake manifold geometry could be evaluated by the differences in the in-cylinder flow characteristic, turbulence, and spark plug gap flow between cylinders.

Therefore, in this study, the two kinds of intake manifold geometry which have different flow path were used to investigate the cylinder-to-cylinder variation in GDI engine system using computational fluid dynamic (CFD) program CONVERGE v2.4. The intake manifold geometries (curved intake manifold^24,25^, straight intake manifold^26,27^) were chosen based on flow path type which conventionally used for internal combustion engines. The simulation results were validated with comparing the particle image velocimetry (PIV) results. The cylinder-to-cylinder variation was analyzed from the perspective of volumetric efficiency, early tumble development process, turbulent kinetic energy, and the spark plug gap velocity near TDC which were important parameters in GDI engine combustion process. Furthermore, to enhance the in-cylinder flow and turbulent kinetic energy near TDC of straight manifold, the influence of intake manifold curve radius was investigated.

## Methods

### Optically accessible engine

An optically accessible 2-cylinder engine was used for this experiment. A transparent quartz was used to visualize the in-cylinder flow using the particle image velocimetry (PIV) technique. To secure the quality of PIV raw image, the oil free system was replaced as a bearing system, so the raw images were not disturbed by the oil in the cylinder. And the optically accessible engine was operated by AC motor of 37 kW, while controlled by using NI compact RIO system. The cylinder bore was 75.6 mm and the stroke was 83.38 mm. The detailed specifications of the optically accessible engine are presented in Table 1.

**Table 1:** Engine specification.

Compression ratio	14.1
Bore	75.6 mm
Stroke	83.38 mm
Conrod length	147.8 mm
Displacement (2 cylinders)	748.5 cc
Rated RPM	6300 rpm

**Figure 1:**
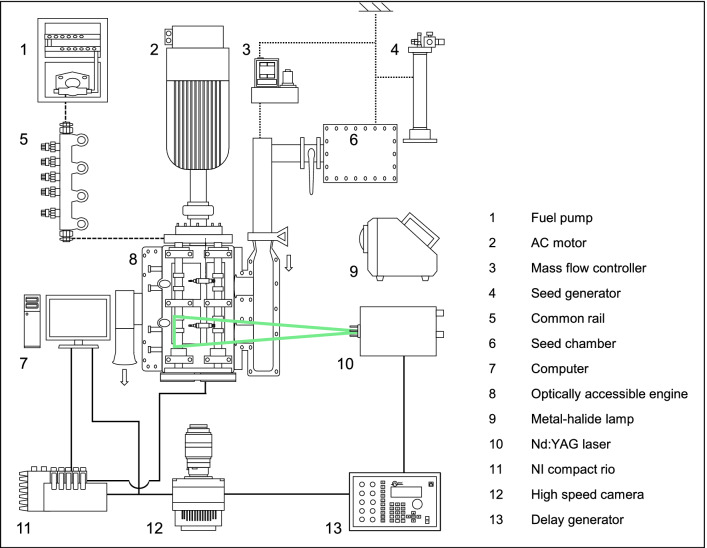
Schematic diagram of the engine operating system.

Figure 1 shows the schematic diagram of the engine operating system. The intake air was controlled by a mass flow meter and an intake chamber was used to reduce the fluctuation of the intake flow from the mass flow meter to the intake manifold. Engine data was acquired by using an encoder that was attached to the crank shaft directly, NI DAQ, and NI compact RIO.

Additionally, to visualize the intake flow in the manifold, a transparent manifold made as an acrylic was used. It was difficult to visualize the intake flow in the manifold by using PIV imaging technique because of attached seeds on the surface of the manifold. Therefore, instead of the seeds for PIV, a BnB-1 middle fog was supplied into the cylinder by using a fog machine to visualize the intake flow in manifold. In experiments of visualizing manifold, seed chamber was used as a fog chamber. Inflowing fog images were captured by using a high-speed camera with a metal-halide lamp in steady state operation. Intake valves were fully opened and piston was eliminated to realize a steady state of inflowing air. And intake air controlled by mass flow controller was continuously supplied into the cylinder. After intake air flow rate was stabilized, a fog was supplied additionally. Visualizing the intake flow in the manifold using a fog cannot show the exact quantitative data. However, the qualitative analysis by observing structure of intake flow in the manifold can be done. Moreover, more detailed analysis can be performed with additional simulation.

### Particle image velocimetry (PIV) system

**Figure 2:**
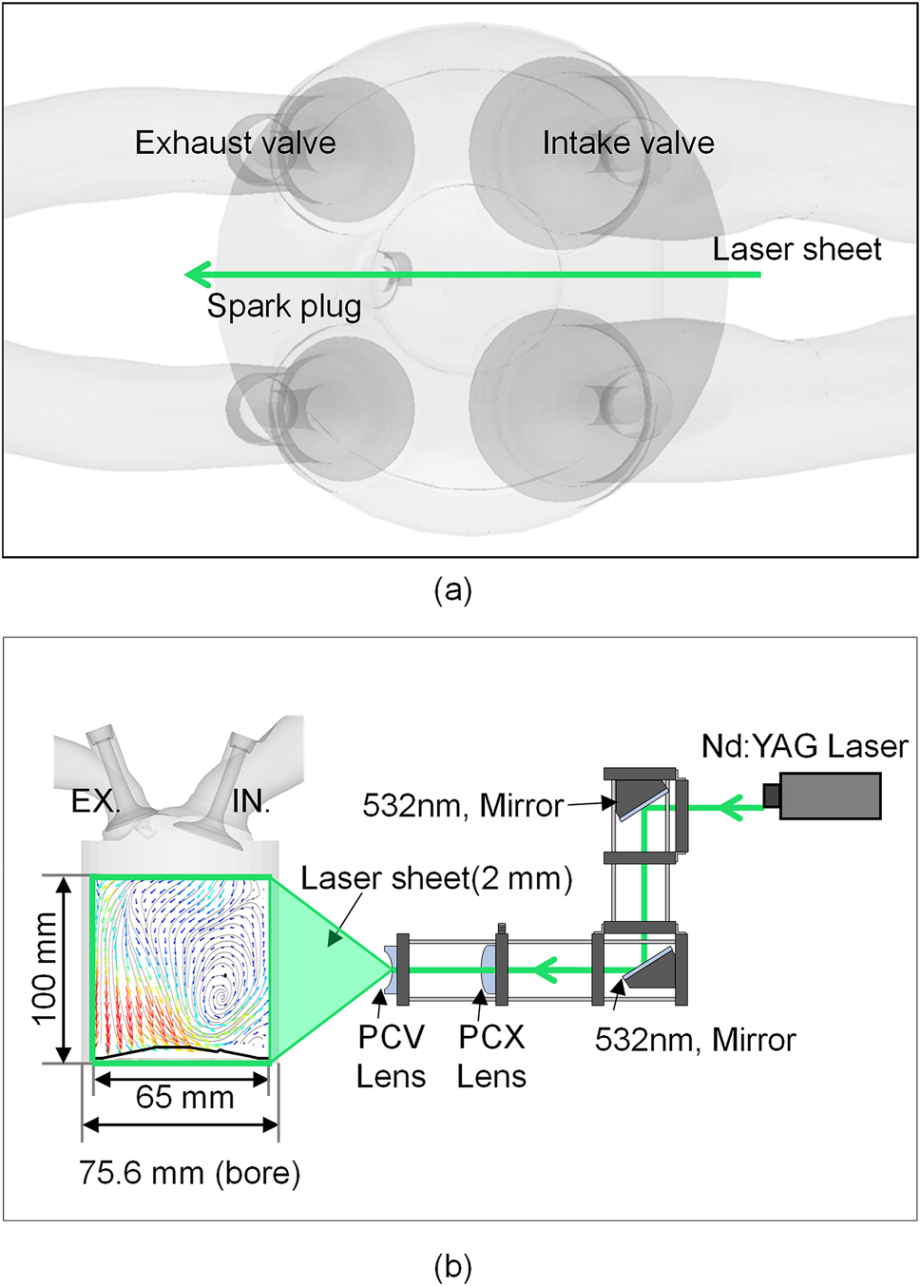
PIV system configuration and image plane: (**a**) top-view and (**b**) front-view.

**Figure 3:**
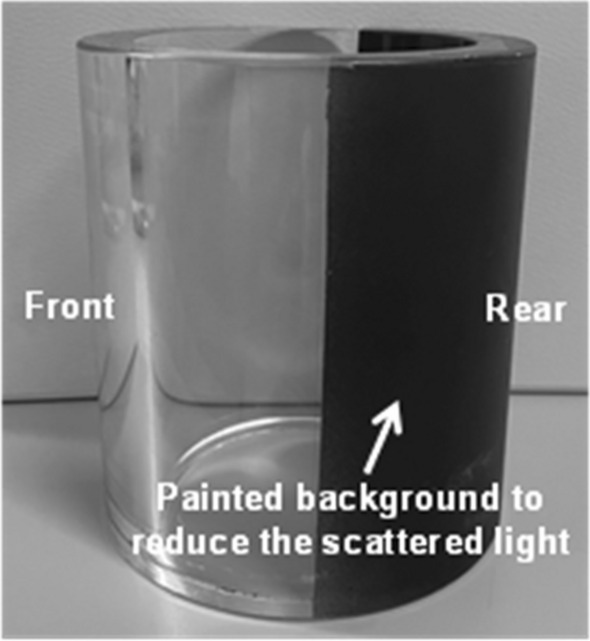
Quartz cylinder used to PIV experiment.

A schematic drawing of the PIV system and image plane is shown in Fig. 2. A laser sheet was generated by a Nd:YAG pulse laser (double-cavity, power: 26 mJ, wavelength: 532 nm). And laser sheet thickness was controlled 2 mm to minimize the refractive index deviation. Planar convex and concave lenses were used to expand a laser beam entering the engine cylinder from the intake valve to the exhaust valve. To minimize the distortion of the laser, the laser was irradiated to the center of the cylinder in the normal direction of the quartz surface. Also, considering the laser scattering inside and outside the cylinder by reflection of laser light, the back of the quartz e.g. opposite side of the camera position was painted black as shown in Fig. 3. Moreover, preventing the distortion due to the curved glass cylinder, a high-speed camera focused on regions near the cylinder axis^28,29^. And the minimized distorted PIV images using camera and lenses operated with a depth focus larger than the image plane thickness to provide focused particles with high signal intensity. The image plane had a width of 65 mm and a height of 100 mm except for the pent roof region. A hollow sphere glass which had a diameter of 10 m$\upmu$ and density of 1.0 g/cc*,* was used as a tracer of the intake flow. Particles were supplied through the particle mixing chamber connected to the particle generator. The illuminated particles were captured by a high-speed camera (Phantom, VEO 710) with a Nikon 105 mm macro lens. A delay generator was used to synchronize the high-speed camera shutter timing and the laser oscillation timing. The PIV images were processed using MATLAB software (PIV lab). Additional functions, such as calculating the tumble ratio, turbulent kinetic energy, and vortex core position, were added to analyze the characteristics of the in-cylinder flow quantitatively. To detect the particle displacement, FFT cross-correlation and the Gauss 2 × 3-point algorithm were used. The mean vector fields were derived from 40 images recorded at each crank angle position and each engine speed. The first correlation employed a 64 × 64 interrogation area with 50% overlap. A second interrogation area had 32 × 32 pixels. In this experiment, the time interval at the intake stroke was set as 0.1 ms. PIV experiment for validation was conducted in engine speed of 1500 rpm, intake pressure of 1.0 bar with the curved manifold type. And captured image from cylinder 1 was used.

### Reliability of PIV experiment results

To evaluate the reliability of the PIV experiment results and the seeding particles, the analysis based on the Stokes number as a criterion of tracking capability of tracer in PIV measurement was conducted in this section. In general, it is considered that seeds follow flow well under Stokes number << 1. The Stokes number is a dimensionless number characterising the behavior of particles suspended in flow, and is defined as Eq. (1)

1 $$Stk= \frac{{\tau }_{p}}{\tau }$$

where ${\tau }_{p}$ is a particle time constant and $\tau$ is a characteristic time scale for the flow of interest. Therefore, the Stokes number was considered in three different time scales which were the engine time scale, mean tumble vortex time scale, and turbulent turn-over time scale. The Stokes number of PIV system in this study was calculated as the previous studies^30,31^.

Because the hollow glass seed is spherical, the particle time constant can be calculated as Eq. (2) where ${\rho }_{p}$ is particle density, ${d}_{p}$ is particle diameter, and ${\mu }_{f}$ is viscosity of the fluid.

2 $${\tau }_{p}= {\rho }_{p}{d}_{p}^{2}/18{\mu }_{f}$$

The viscosity of fluid was used at the atmospheric pressure and room temperature of 298.15 K. And ${\tau }_{p}$ was approximately $3.0 \times {10}^{-4}\mathrm{ s}$ in this PIV system.

The engine time scale was calculated as Eq. (3) where $S$ is the stroke and $\overline{{S}_{p}}$ is the mean piston speed.

3 $${\tau }_{e}=S/\overline{{S}_{p}}$$

It had value of $2.0 \times {10}^{-2} \mathrm{s}$ at engine speed of 1500 rpm, and corresponding stokes number is $1.5\times {10}^{-2} \mathrm{s}$. Therfore, the particles can follow the variation of the largest flow structure. And the mean tumble vortex turn-over time scale (${\tau }_{tv}$) with Eq. (4), where ${\omega }_{e}$ is engine crank rotation speed and $TR$ is tumble ratio, was $3.2\times {10}^{-3} \mathrm{s}$.

4 $${\tau }_{tv}=1/{(\omega }_{e}\bullet TR)$$

As a result, Stokes number was derived as a value of $9.4\times {10}^{-2} \mathrm{s}$, which means that the particle can track the large-scale tumbling structure. And for more smaller time scale which is the tubulent turn-over time scale (${\tau }_{t})$ can be calculated as Eq. (5), where $L$ is large-eddy length scale and ${u}^{^{\prime}}$ is turbulent velocity scale values.

5 $${\tau }_{t}=L/{u}^{^{\prime}}$$

According to Lumley^32^, ($L$, ${u}^{^{\prime}}$) can be estimated $(L$,${u}^{^{\prime}})=(B/6, \overline{{S}_{p}}$) at bTDC 180 deg and $(L$, ${u}^{^{\prime}})=(B/6 ,\overline{{S}_{p}}/2$) at bTDC 90 deg in engine system where $B$ is the bore. Therefore, ${\tau }_{t}$ had the value of $3.0\times {10}^{-3} \mathrm{s}$ at bTDC 180 deg and of $3.8\times {10}^{-3} \mathrm{s}$ at bTDC 90 deg. It came to the Stokes number of $9.9\times {10}^{-2} \mathrm{s}$ at bTDC 180 deg and of $7.8\times {10}^{-2} \mathrm{s}$ at bTDC 90 deg. This result confirmed that the particle can track the large-scale turbulent velocity fluctuations.

Thus, the PIV system in this study could present the flow characteristic from the relatively large scale of engine dimension to the small scale of turbulence.

### Vector field computation and analysis

To analyze the in-cylinder flow quantitatively, the tumble ratio and turbulent kinetic energy were computed using the MATLAB program. In this experiment, the tumble ratio and turbulent kinetic energy were calculated from an image plane except for the pent roof region. The details about deriving the tumble ratio and turbulent kinetic energy are specified as follows^33^:

6 $$  Tumble~Ratio = ~\frac{{\mathop \sum \nolimits_{{i = 1}}^{n} \mathop \sum \nolimits_{{j = 1}}^{m} \left( {\overset{\lower0.5em\hbox{$\smash{\scriptscriptstyle\rightharpoonup}$}}{{r_{{i,j}} }}  - \overset{\lower0.5em\hbox{$\smash{\scriptscriptstyle\rightharpoonup}$}}{{r_{c} }} } \right) \times \overset{\lower0.5em\hbox{$\smash{\scriptscriptstyle\rightharpoonup}$}}{{V_{{i,j}} }} }}{{\omega \mathop \sum \nolimits_{{i = 1}}^{n} \mathop \sum \nolimits_{{j = 1}}^{m} \left( {\overset{\lower0.5em\hbox{$\smash{\scriptscriptstyle\rightharpoonup}$}}{{r_{{i,j}} }}  - \overset{\lower0.5em\hbox{$\smash{\scriptscriptstyle\rightharpoonup}$}}{{r_{c} }} } \right) \cdot \left( {\overset{\lower0.5em\hbox{$\smash{\scriptscriptstyle\rightharpoonup}$}}{{r_{{i,j}} }}  - \overset{\lower0.5em\hbox{$\smash{\scriptscriptstyle\rightharpoonup}$}}{{r_{c} }} } \right)}}  $$

$$ \overset{\lower0.5em\hbox{$\smash{\scriptscriptstyle\rightharpoonup}$}}{{r_{{i,j}} }}  - \overset{\lower0.5em\hbox{$\smash{\scriptscriptstyle\rightharpoonup}$}}{{r_{c} }}  $$: Distance of a given location with indices ($$i,j$$) to the center of rotation,

$$ \overset{\lower0.5em\hbox{$\smash{\scriptscriptstyle\rightharpoonup}$}}{{V_{{i,j}} }}  $$: Velocity in that location, and

$\omega$: Engine’s angular (crank) speed.

The tumble ratio is defined as the ratio of dividing an angular velocity of flow by an angular velocity of engine rotation. Although the real in-cylinder flow has a three-dimensional structure, the main motion of tumble flow is in a vertical cross section. Therefore, two-dimensional PIV data measured from an image plane can be used to analyze the characteristics of the tumble flow as follows^34^:

7 $$Turbulent kinetic energy=0.5 \times (\stackrel{-}{{{(u}^{^{\prime}})}^{2}}+\stackrel{-}{{{(v}^{^{\prime}})}^{2}})$$

${u}^{\mathrm{^{\prime}}}$: Total radial velocity of turbulent vector field in an image plane, and.

${v}^{^{\prime}}$: Total axial velocity of turbulent vector field in an image plane.

Turbulent kinetic energy was computed based on the fluctuation component of the turbulent vector field. The turbulent vector field was calculated by subtracting the mean flow vector field in the flow vector field of each cycle.

### Computational details

The engine simulations were carried out using CONVERE v2.4 as the three-dimensional CFD program.

**Figure 4:**
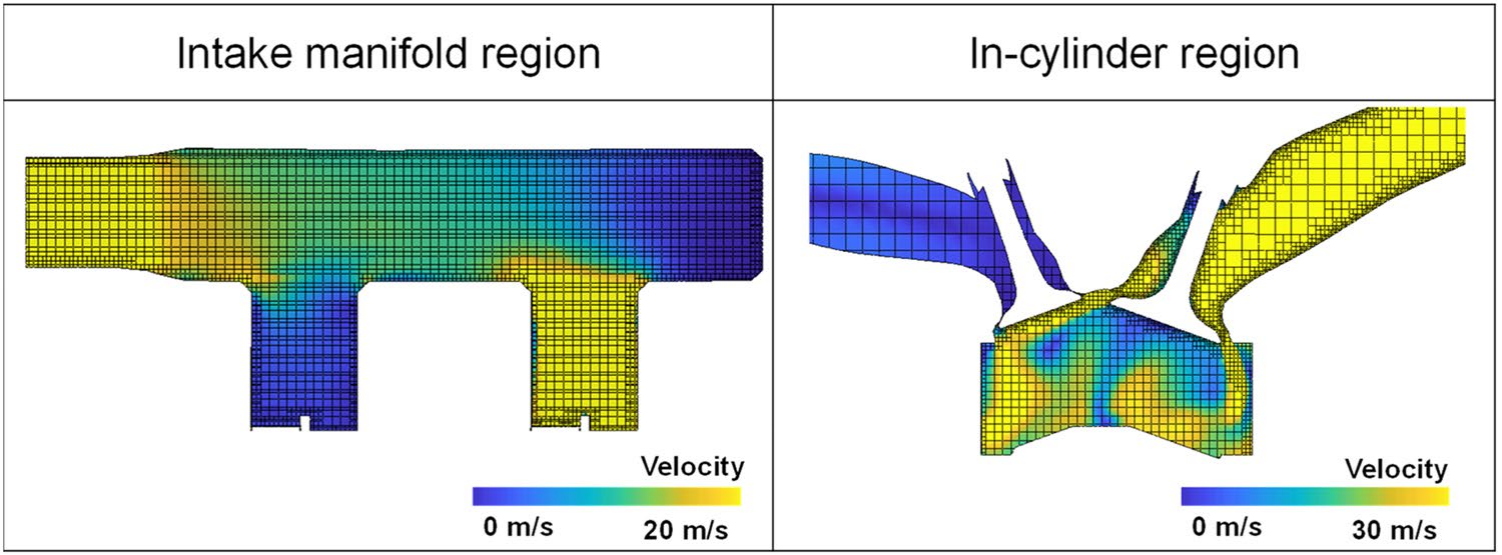
Cut-plane of computational cells at bTDC 300°.

The base grid size of 4.0 mm was applied. An additional grid embedding was set in the in-cylinder region to refine grid to 2.0 mm, in in-cylinder boundary to 1.0 mm, and in the valve and intake port boundary to 0.5 mm. Adaptive mesh refinement with the embedding level of 2 was used to generate more refined cells where the velocity difference between cells higher than 2.5 m/s. Therefore, the meshes were refined in region where the fast flow was formed as shown in Fig. 4. As a result, the maximum total number of about 900,000 cells was formed during simulation time in case of engine speed of 1500 rpm and intake pressure of 1.0 bar.

To investigate the effects of the intake manifold geometry on the in-cylinder flow characteristics, the simulation was conducted in conditions as shown in Table 2. The valve event was presented based on the valve lift of 1 mm and the timing was based on the top dead-center of the compression stroke. The two cylinders were considered to investigate the cylinder-to-cylinder variation.

**Table 2:** Simulation condition.

Engine speed	1500 rpm	1500 rpm	2000 rpm	3000 rpm
Intake manifold inlet pressure	1.0 bar*	1.3 bar	1.0 bar	1.0 bar
IVO	bTDC 332°			
IVC	bTDC 114°			
EVO	aTDC 152°			
EVC	aTDC 370°			

**Figure 5:**
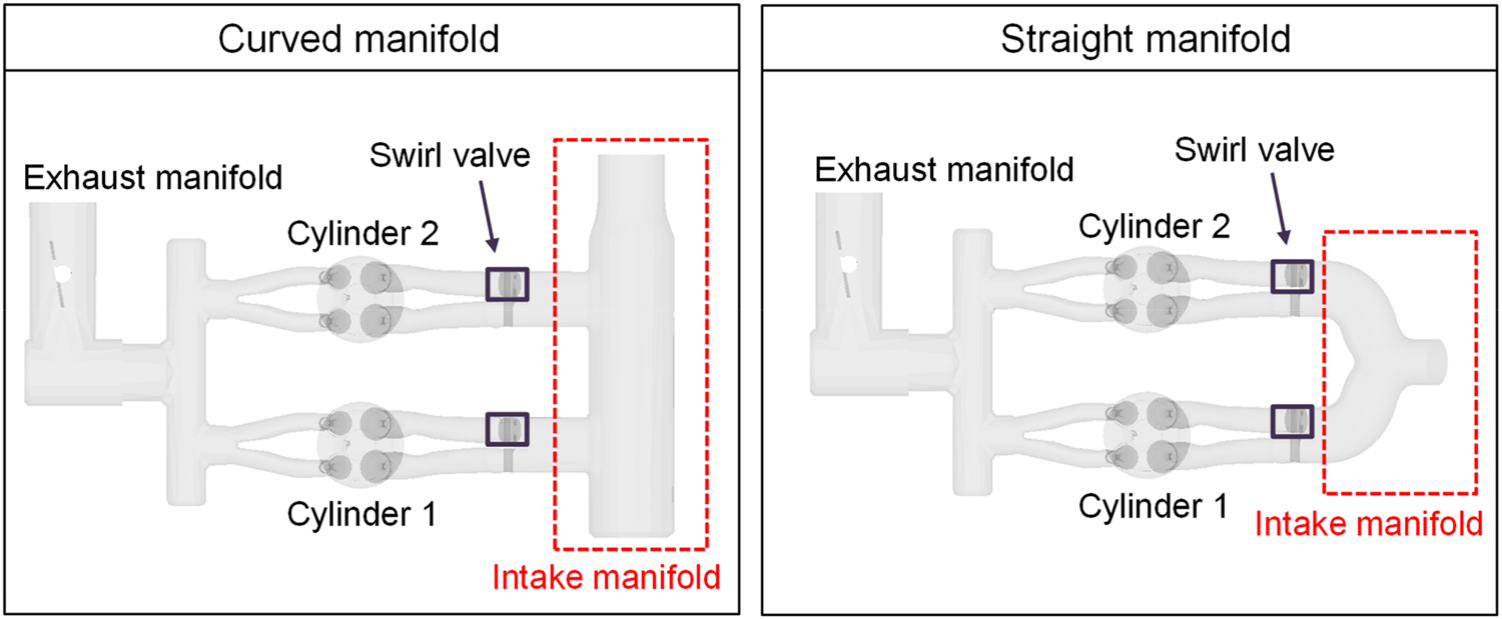
Computational domain for simulation.

Initially, the two types of intake manifold geometry were used to investigate the effects of the intake manifold geometry on cylinder-to-cylinder variation in intake manifold and cylinders (Fig. 5). The computation domain has been set including the intake manifold, two cylinders, and exhaust manifold for multi-cylinder calculation. These were basic designs for the intake manifold. The curved manifold consisted of one flow path supplied to each intake port, and the flow direction at the manifold inlet and the port direction were vertical. The straight manifold had a shape in which the flow direction at the manifold inlet and the port direction were in-line, and the flow path split when it was supplied to each intake port.

**Figure 6:**
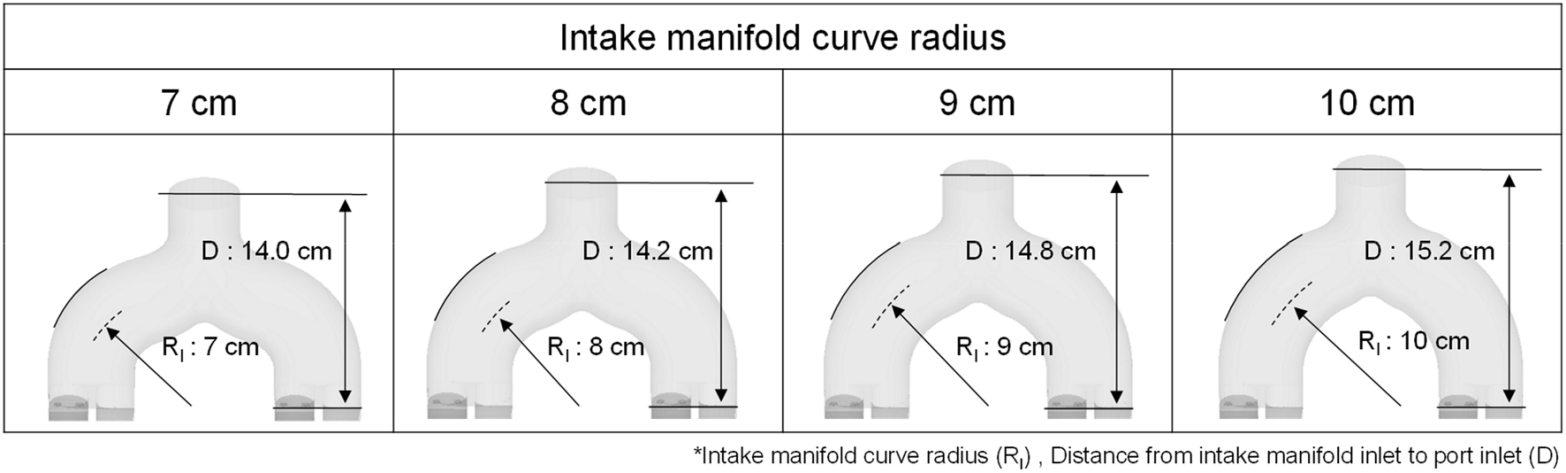
Intake manifold geometry for evaluating the effect of intake manifold curve radius.

Based on straight manifold design, the influence of the intake manifold curve radius on the intake process through the intake manifold and intake port was evaluated. The intake manifold geometry for different intake manifold curve radius is shown in Fig. 6.

The cylinder-to-cylinder variation was investigated with respect to the volumetric efficiency, early tumble development, turbulent kinetic energy, and spark plug gap velocity. And those results were calculated as follows. The volumetric efficiency was calculated based on the in-cylinder air mass and air density after IVC as follow Eq. (8) where *V*_*d*_ is displacement.

8 $$VE= {m}_{air}/({\rho}_{air} \cdot {V}_{d}).$$

To quantify the in-cylinder turbulence level, the tumble ratio and turbulent kinetic energy were used. The tumble ratio was defined as the ratio of the angular speed of the flow about the center of mass in the tumble direction, $\omega$, to the angular speed of the crank shaft, ${\omega }_{crank shaft}$, as shown in Eq. (9) as follows:

9 $$tumble ratio= \omega/{\omega }_{crank shaft}.$$

The turbulent kinetic energy, $k$, was defined as half of the trace of the stress tensor as shown in Eq. (10) where $\widetilde{{u}_{i}}$ is defined as Eq. (11).

10 $$k=\frac{1}{2}\widetilde{{u}_{i}{u}_{i}}$$

11 $$\widetilde{{u}_{i}}\equiv \overline{\rho {u }_{i}}/\overline{\rho}.$$

For the turbulent kinetic energy of the region, the mass averaged turbulent kinetic energy was used.

**Figure 7:**
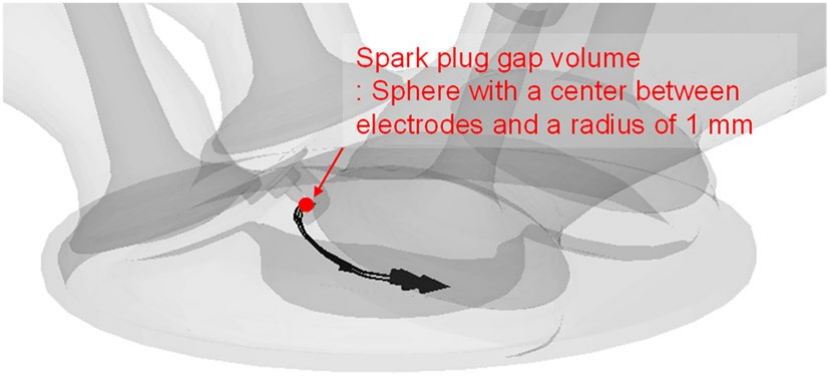
Definition of spark plug gap volume.

The spark plug gap velocity near TDC was calculated in spark plug gap volume as defined in Fig. 7. The mean spark plug gap velocity in this volume presented the spark plug gap velocity ($${V}_{spark})$$ and it was calculated as in Eq. (12).

12 $${V}_{spark}= \int {v}_{cell}dV/\int dV.$$

The volume averaged velocity was calculated, and each cell volume in spark plug gap was considered.

### Computational model

To predict the turbulent characteristics of the in-cylinder flow effectively, a proper turbulence model is required. In this study, the RNG k–ε model^35,36^ was used for closure, which has shown high accuracy in many prior internal combustion engine simulation studies^37–40^.

### Validation

**Figure 8:**
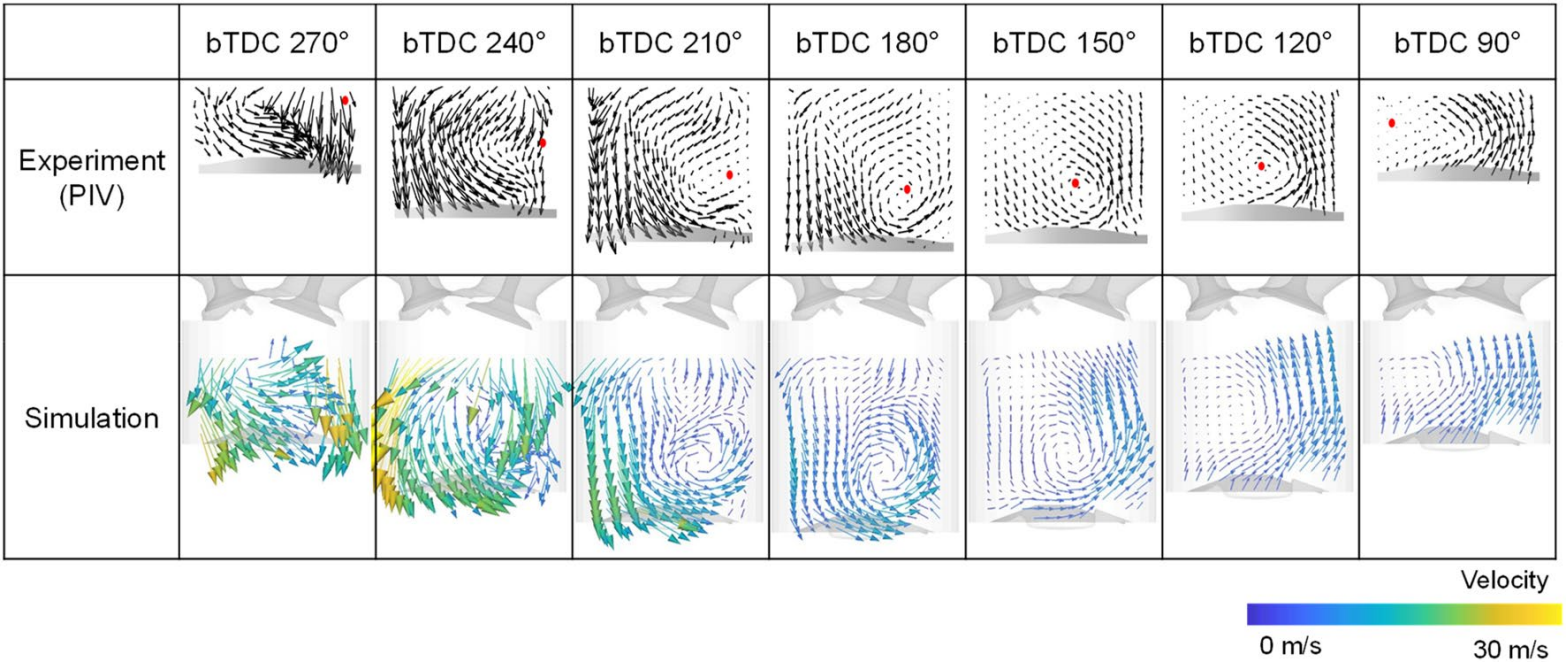
Comparison of the cross-sectional velocity distribution between the experiment and simulation.

**Figure 9:**
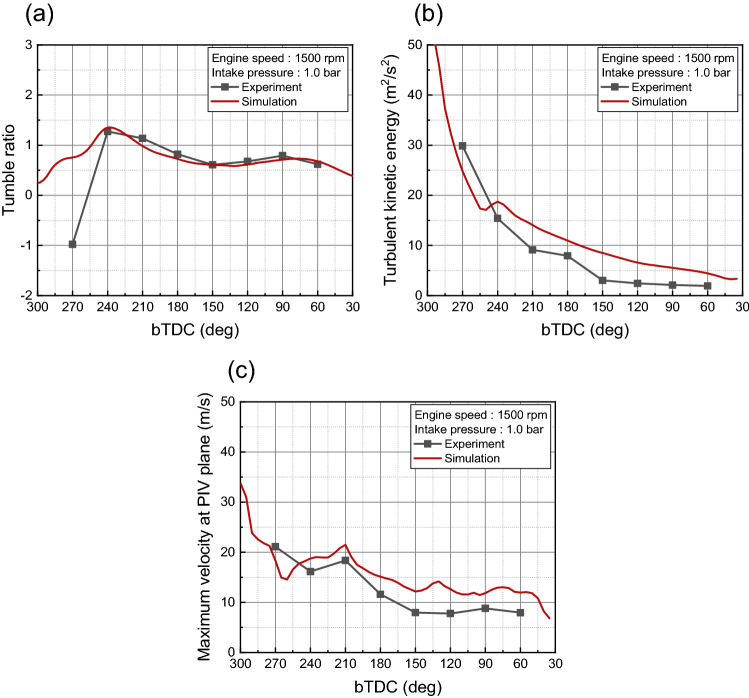
Comparison quantitative data between experiment and simulation: (**a**) tumble ratio, (**b**) turbulent kinetic energy, (**c**) maximum velocity.

To confirm the consistency of the experiment and simulation, validation was conducted. For the validation of the in-cylinder flow characteristics, PIV results and simulation results using the curved intake manifold were compared at an intake process are compared in Fig. 8. The simulation was conducted under same condition with experiment, i.e., engine speed of 1500 rpm, intake pressure of 1.0 bar. The planes were clipped at the center of the cylinder. And the length scale of the arrow proportional to the velocity magnitude was the same between the experiment and simulation. The flow supplied from the intake valve at the beginning of the intake process was directed toward the exhaust valve, hit the piston to form a tumble, and then went through a compression process to form a flow rising toward the intake valve in the experimental results. The simulation results had a similar trend in velocity distribution. Furthermore, the qualitative data were compared in Fig. 9 with respect to the tumble ratio, turbulent kinetic energy, and maximum velocity in PIV plane. Those results selected for validation because they could be represented the in-cylinder flow characteristics. There was a difference between the experimental and simulation results for the tumble ratio in bTDC 270°, at which the flow had a strong turbulence due to fast intake flow (Fig. 9a). The tumble ratio differentiation in bTDC 270° was caused by the strong disturbance and the narrow ROI of experiment as can be seen in Fig. 8. However, the simulation results showed high accuracy in the subsequent results. Although the differentiation between the PIV experiment and simulation results of the turbulent kinetic energy and maximum velocity were increased in compression process, the overall trend was well represented by simulation (Fig. 9b,c).

**Figure 10:**
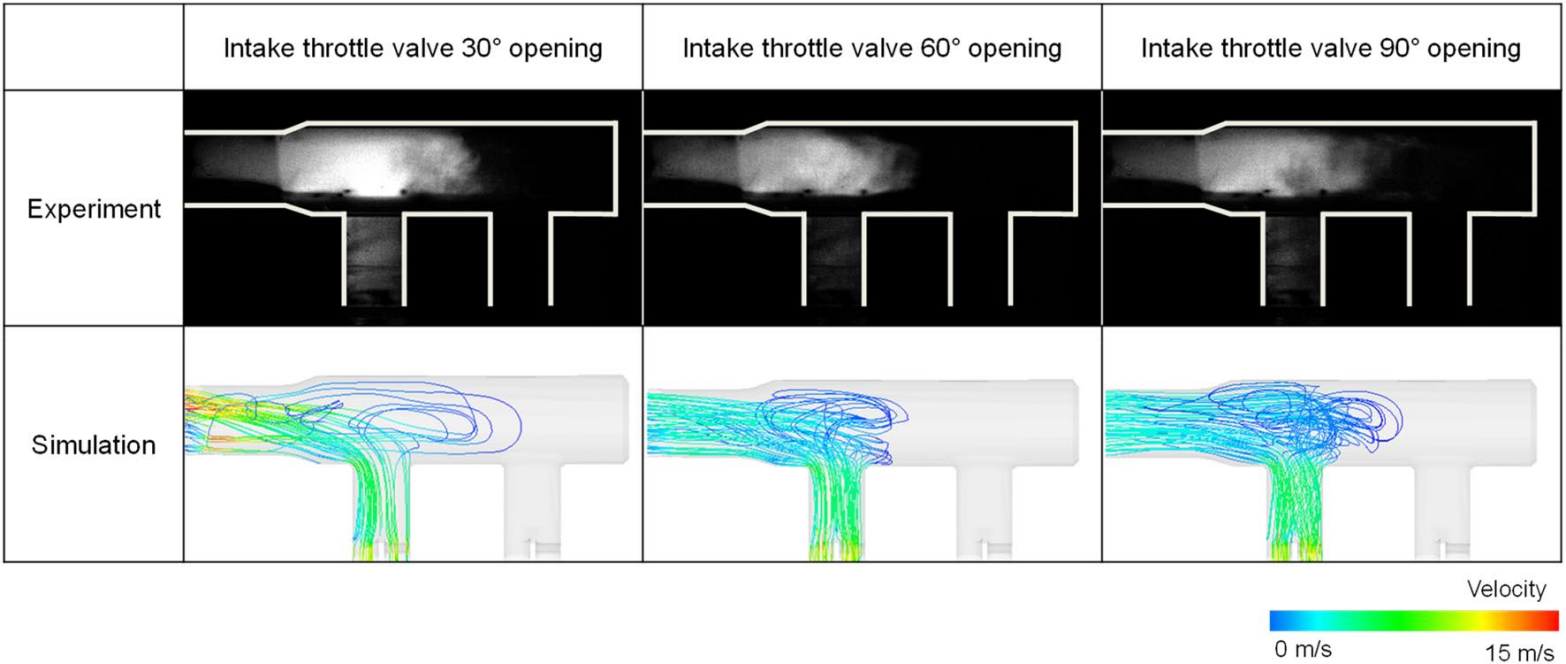
Image comparison of the intake manifold flow in steady-state conditions between the experiment and simulation.

For the flow in the intake manifold, the simulation result was compared with the Mie-scattering experimental results under steady-state conditions in which cylinder 2 was open and the inlet air flow rate was 8 g/s. The Mie-scattering was conducted using a metal-halide lamp as a light source and BnB-1 middle fog as a flow tracer. Three conditions were used with varying intake throttle valve opening angles. The image of the distribution of fog in the steady-state experiment demonstrated the stream-lines of the flow from intake manifold inlet to the cylinder. Thus, the simulation results of stream-lines that were conducted under the same condition of the experiment were compared with the Mie-scattering image using fog. As a result of validation of the intake manifold flow, the flow travel distance and distribution in the intake manifold showed similar results with various intake throttle valve opening angles (Fig. 10).

## Results and discussion

### Performance of curved manifold and straight manifold

One of the crucial factors in enhancing intake manifold performance is reducing cylinder-to-cylinder variation for stable combustion between cylinders. Thus, to evaluate the performance of the curved manifold and straight manifold, the intake process was investigated from the aspect of the cylinder-to-cylinder variation. And further, the tumble motion was investigated. Because strong tumble during intake process is important for GDI engine system to enhance the spray atomization preventing wall wetting and promoting evaporation of fuel droplets^41^.

**Figure 11:**
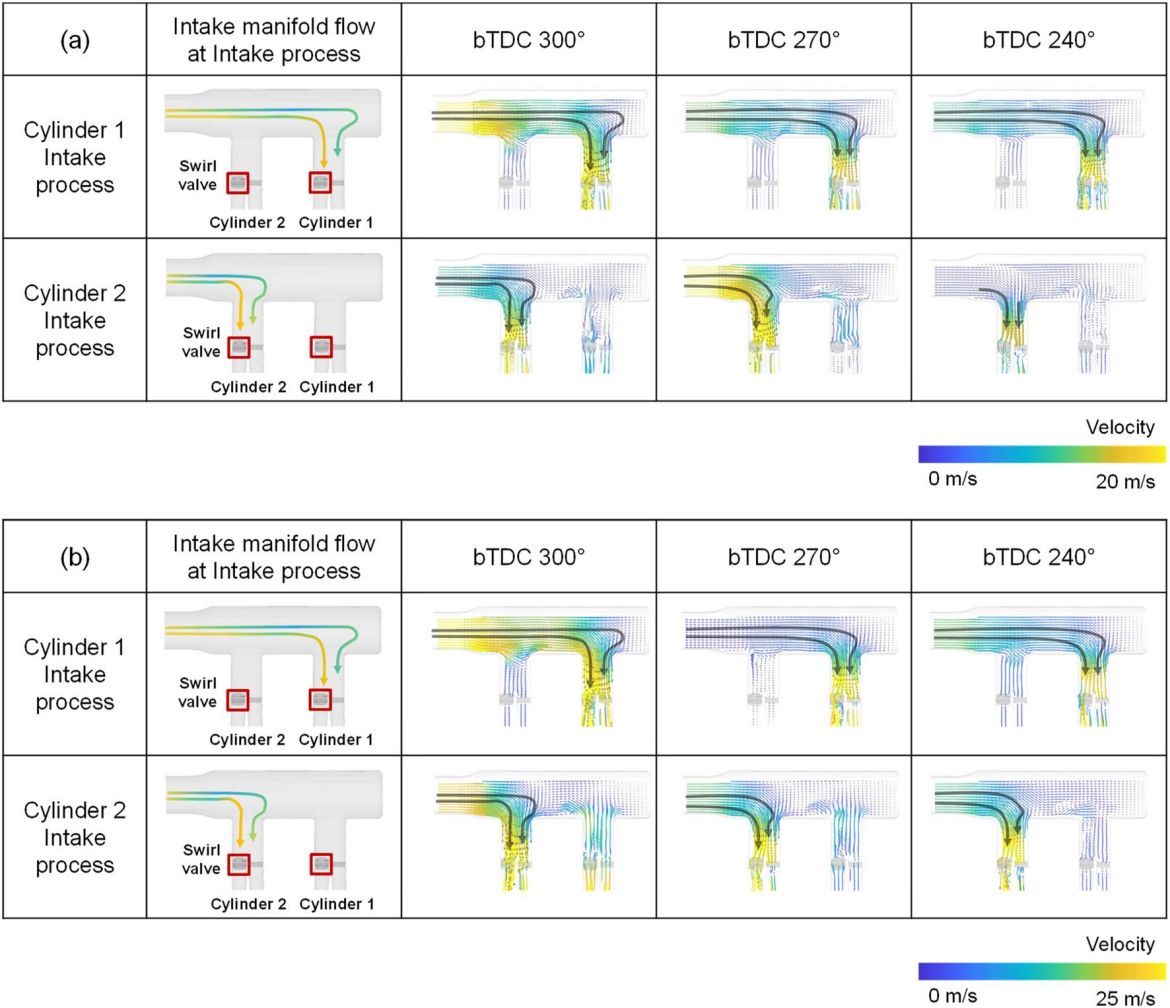
Cross-sectional velocity distribution for the curved manifold during intake process: (**a**) engine speed: 1500 rpm, intake pressure: 1 bar, (**b**) engine speed: 2000 rpm, intake pressure: 1 bar.

To understand the cylinder-to-cylinder variation due to the intake manifold geometry, first, the intake manifold flow development during intake process was analyzed. The curved intake manifold had a shape in which the direction of the air flow supplied from the manifold inlet and the direction of the port were vertical. The distance from the intake manifold inlet to the intake port inlet differed between cylinders 1 and 2 due to the structural features. Since the position of the intake port of cylinder 2 was located closer to the intake manifold inlet, the velocity of the flow at the port inlet of cylinder 2 was faster and the momentum was larger during intake process (Fig. 11). The velocity distribution inside the intake manifold showed a maximum velocity at the manifold inlet, so that the induction speed at the port close to the manifold inlet, that is, the port where the swirl valve is located, appeared fast in intake process of both cylinders.

**Figure 12:**
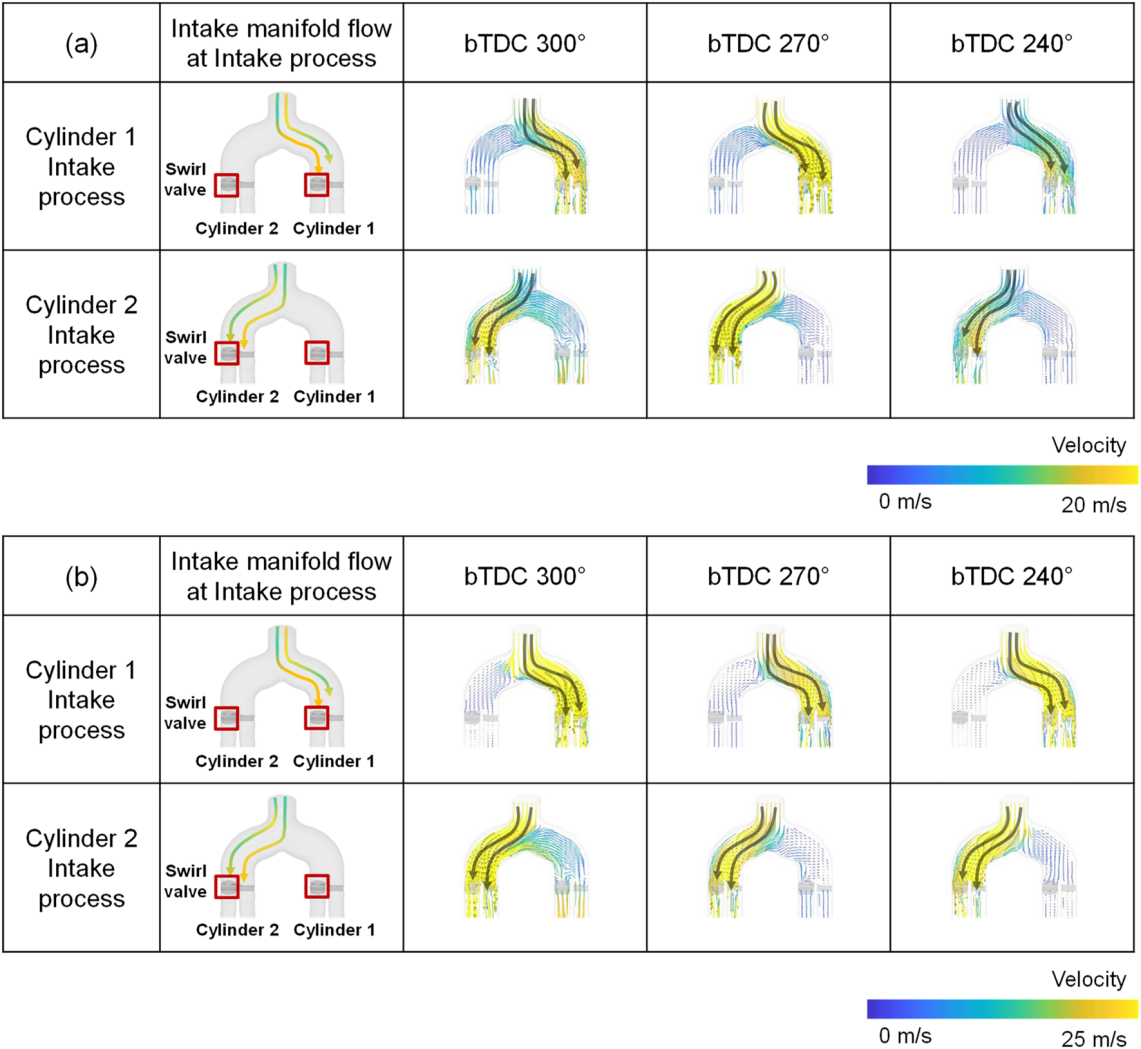
Cross-sectional velocity distribution for the straight manifold during intake process: (**a**) engine speed: 1500 rpm, intake pressure: 1 bar, (**b**) engine speed: 2000 rpm, intake pressure: 1 bar.

On the other hand, the straight manifold was in line with the direction of the flow supplied from the inlet of the intake manifold and the direction of the intake port. There was a curved path near the intake port inlet. It resulted in a faster flow velocity towards the inner intake port than towards the outer intake port (Fig. 12)^42^. The straight manifold has a symmetrical shape compared to the curved manifold. However, there was a difference between cylinders in the existence of a swirl valve at the inner port where flow was strengthened.

**Figure 13:**
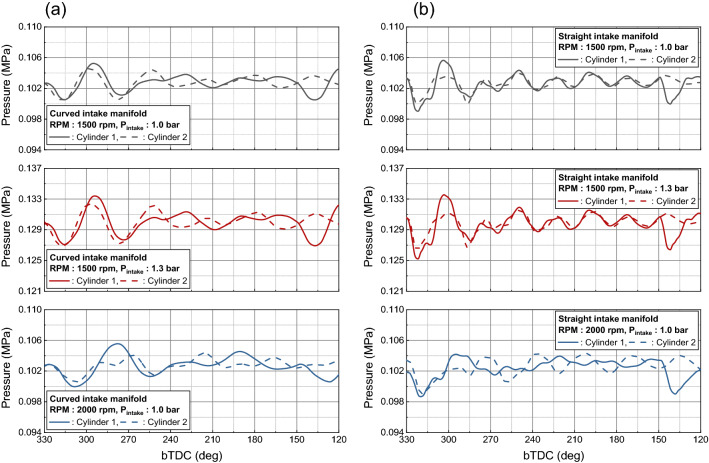
Pressure fluctuation in intake manifold for different intake manifold: (**a**) curved manifold, (**b**) straight manifold.

Figure 13 shows the trend of pressure fluctuation for different intake manifold geometry during the intake process. The curved intake manifold cases appear a difference in pressure fluctuations depending on the cylinder during the intake process for all cases. And pressure of cylinder 1 lagged behind cylinder 2 due to the difference in distance from the intake manifold inlet. On the other hand, in the straight manifold, fluctuations occur during the intake process of cylinder 2 at 2000 rpm, causing a difference between cylinders. This is because there was more suction in cylinder 2 due to the absence of the swirl valve on the inner port where the high velocity was formed, and this effect increased as the engine speed increased.

**Figure 14:**
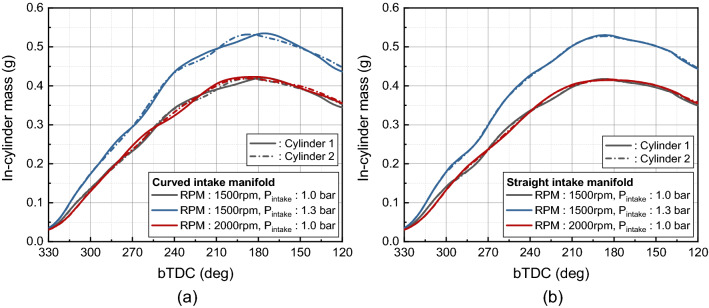
In-cylinder charged mass of each cylinders: (**a**) curved intake manifold, (**b**) straight intake manifold.

**Figure 15:**
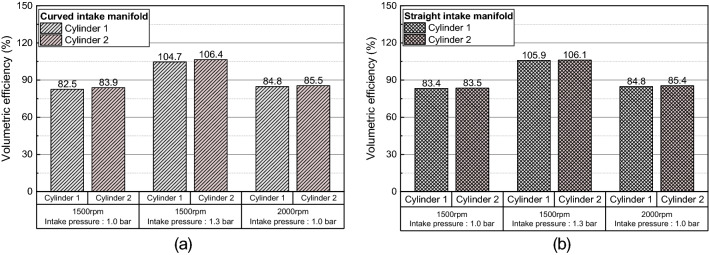
Volumetric of each cylinders: (**a**) curved intake manifold, (**b**) straight intake manifold.

Figure 14 shows the charged mass in cylinder for different intake manifold. In the case of curved manifold, a variation of the charged mass flow rate occurred between cylinders during the intake process due to the difference in distance from the manifold inlet to the port of each cylinder (Fig. 14a). Since the distance from the manifold inlet to cylinder 2 is shorter than the distance to cylinder 1, after the intake process, the charged mass in the cylinder was greater in cylinder 2. As a result, the volumetric efficiency of cylinder 2 was greater than that of cylinder 1 for curved intake manifold under all conditions (Fig. 15a). In the case of a straight intake manifold, the charged mass flow rate during the intake process tended to be almost the same for cylinder 1 and cylinder 2 (Fig. 14b). After the intake process, the charged mass in cylinder 2 appeared slightly greater. This result can also be seen from the volumetric efficiency results for cylinders in Fig. 15b. It resulted from the swirl valve located on one side of the intake port. In the straight intake manifold, the flow to the inner port has strengthened during the intake process for both cylinders, but for cylinder 1, the swirl valve has located at the inner port, preventing flow.

In both intake manifold geometry, volumetric efficiency of cylinder 2 was greater. In the curved intake manifold, the difference in the distance between the intake port and the manifold inlet resulted in a maximum 1.7% of the cylinder-to-cylinder variation. On the other hand, in the straight manifold, the presence or absence of a swirl valve in the inner port where the flow is strong resulted in a maximum 0.6% of the cylinder-to-cylinder variation. As shown in Table 3, the cylinder-to-cylinder variation of volumetric efficiency from the asymmetry of the curved intake manifold was greater than the cylinder-to-cylinder variation of volumetric efficiency from the existence of the swirl valve in port. Comparing the effect of the intake manifold geometry on the volumetric efficiency variation between cylinders, the straight manifold geometry with a symmetrical shape had the advantage of reducing the cylinder-to-cylinder variation of volumetric efficiency.

**Table 3:** Difference of volumetric efficiency between cylinders for different intake manifold.

Simulation condition	Curved manifold (%)	Straight manifold (%)
RPM: 1500 rpm, P_intake_ = 1.0 bar	1.4	0.1
RPM: 1500 rpm, P_intake_ = 1.3 bar	1.7	0.2
RPM: 2000 rpm, P_intake_ = 1.0 bar	0.7	0.6

**Figure 16:**
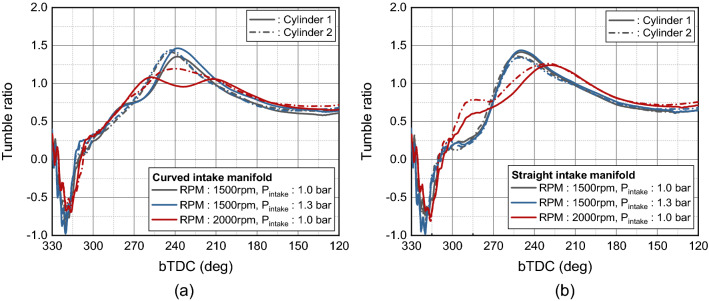
Tumble ratio during intake process: (**a**) curved manifold, (**b**) straight manifold.

In curved manifold, the tumble ratio of cylinder 2 has increased faster because of the better air supply from the intake manifold to cylinder 2 at the early intake process from bTDC 300° to bTDC 240° as shown in Fig. 16a. The pressure fluctuation during intake process of cylinder 1 lagged behind cylinder 2, so the tumble ratio also showed the same trend. Furthermore, at engine speed of 2000 rpm, the effect of the distance difference between the manifold inlet and cylinder was greater, with cylinder 1 having a two peak shape that decreases at bTDC 240°, but cylinder 2 continuously increasing to bTDC 240°. In the straight manifold, the tumble developed similar in cylinders 1 and 2 for engine speed 1500 rpm as shown in Fig. 16b. However, for engine speed 2000 rpm, the tumble generation was promoted in cylinder 2 at the early intake process. This is because the influence of the presence or absence of the swirl valve of the inner port increased as the engine speed increased. With respect to the in-cylinder response of the tumble generation, the overall cylinder-to-cylinder variation for the tumble ratio of curved manifold was more pronounced compared to the straight manifold. And in both intake manifold geometry, the variation of tumble was noticeable as the engine speed increased.

**Figure 17:**
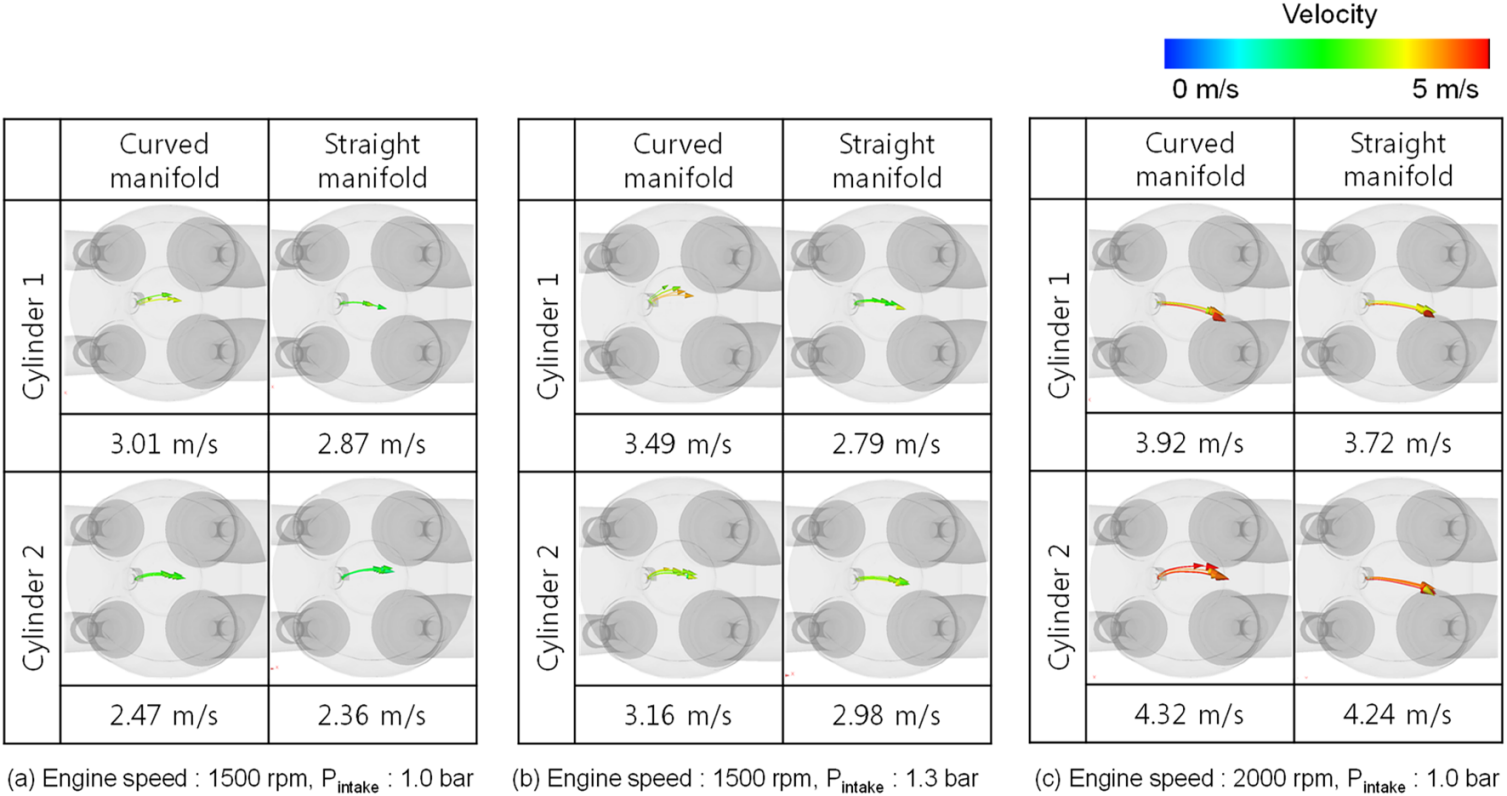
Comparison of spark plug gap velocity between cylinders at TDC.

The spark plug gap velocity is closely associated to the ignition and early flame propagation process, so that combustion properties could be influence by the spark plug gap velocity. The spark plug gap flow intensity and direction for different intake manifold is shown in Fig. 17. The spark plug gap velocity direction which determines the spatial distribution of the early flame had more consistency between cylinders in the straight manifold type. And the velocity difference at TDC was less in the straight manifold type as in Table 4 except of engine speed of 2000 rpm. The spark plug gap velocity difference between cylinders in the straight manifold was reduced 5% at an engine speed of 1500 rpm and intake pressure of 1.0 bar, and 45% at engine speed of 1500 rpm and intake pressure of 1.3bar than the curved manifold. However, in engine speed of 2000 rpm, because of the tumble enhancement in cylinder 2 at the straight manifold type, the spark velocity difference between cylinders was 23% higher than in the curved type.

**Table 4:** Spark plug gap velocity difference between cylinders at TDC.

	Curved manifold (m/s)	Straight manifold (m/s)
Engine speed: 1500 rpm P_intake_: 1.0 bar	0.54	0.51
Engine speed: 1500 rpm P_intake_: 1.3 bar	0.33	0.18
Engine speed: 2000 rpm P_intake_: 1.0 bar	0.40	0.52

Considering the difference of volumetric efficiency, tumble development, and spark plug gap velocity between cylinders, the straight manifold was more effective in engine speed of 1500 rpm, or turbo-charged condition than curved manifold on cylinder-to-cylinder variation.

**Figure 18:**
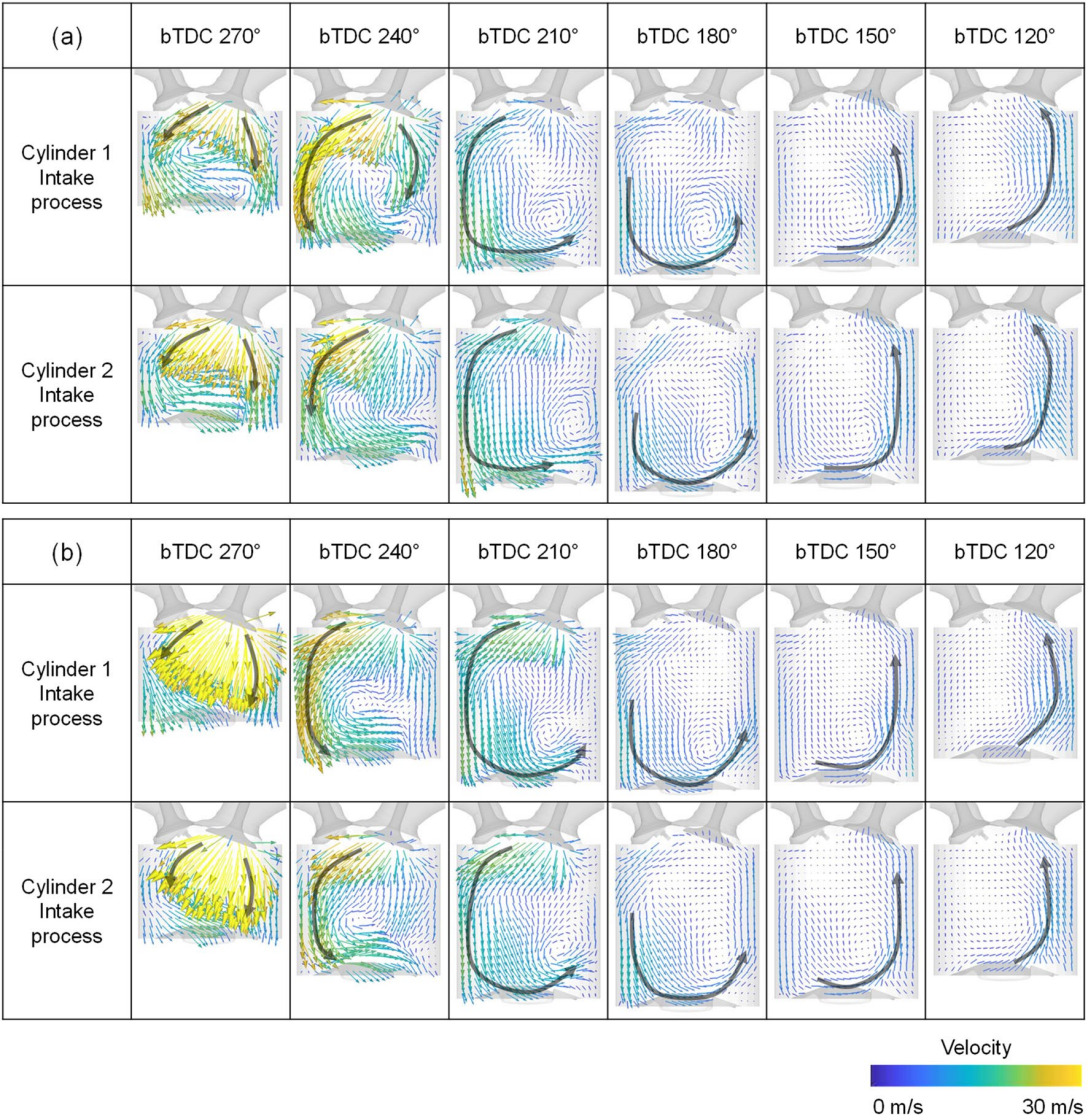
Velocity distribution in tumble plane of cylinder 1 at engine speed of 1500 rpm and intake pressure of 1.0 bar: (**a**) curved intake manifold, (**b**) straight manifold.

**Figure 19:**
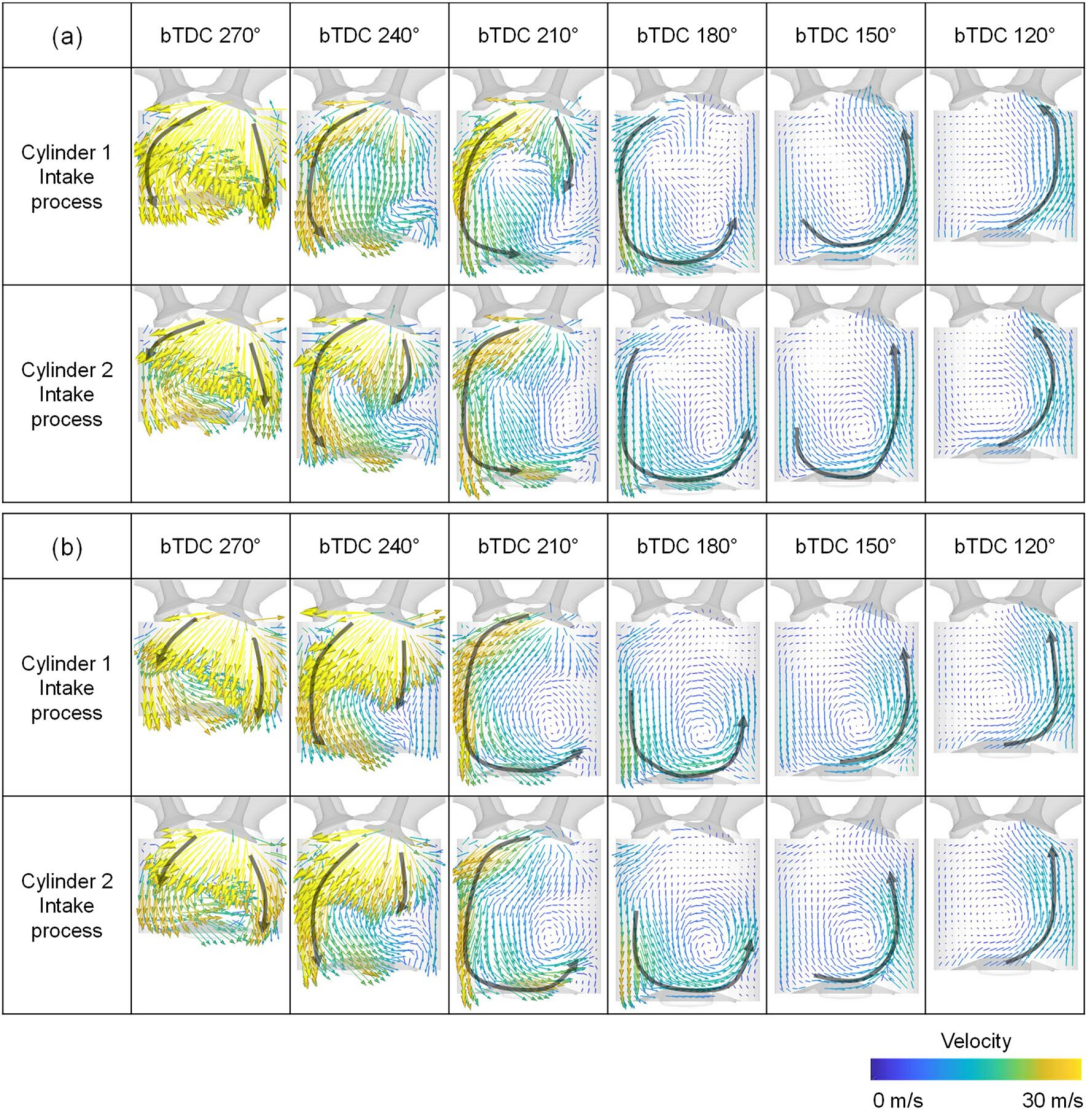
Velocity distribution in tumble plane of cylinder 1 at engine speed of 2000 rpm and intake pressure of 1.0 bar: (**a**) curved intake manifold, (**b**) straight manifold.

Through the velocity distribution in the tumble plane of cylinder 1, it can be seen that the straight intake manifold formed stronger flow intensity during intake process as shown in Figs. 18 and 19. This is because the direction of flow supplied from the intake manifold inlet and the direction of the intake port was in line at the straight intake manifold, so the straight manifold had less flow momentum loss than the curved type.

**Figure 20:**
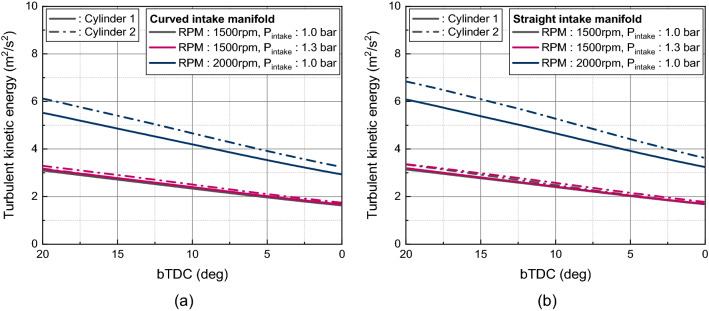
Turbulent kinetic energy near TDC: (**a**) curved manifold, (**b**) straight manifold.

The intensified in-cylinder flow during intake process by the flow supplied by intake port is weakened during the compression process. Through the compression process the flow momentum loss occurred by friction. Additionally, there was flow momentum transition to the turbulent kinetic energy. Because the flow momentum loss in the straight manifold type at intake manifold and port was less than in the curved type, the in-cylinder flow was more strengthened in the straight manifold type, so that the turbulent kinetic energy near TDC was higher in the straight manifold type as in Fig. 20. And this was more pronounced at the higher engine speed of 2000 rpm. At the engine speed of 2000 rpm, the mean turbulent kinetic energy of cylinders was 6.46 m^2^/s^2^ in straight manifold and 5.82 m^2^/s^2^ in curved manifold. This was an 11% increase over the straight manifold.

### Influence of the intake manifold curve radius

The influence of intake manifold curve radius on intake charging process and turbulent characteristics was investigated as an aim of optimization in the straight manifold. The analysis was conducted based on the intake process and in-cylinder motion of cylinder 1.

**Figure 21:**
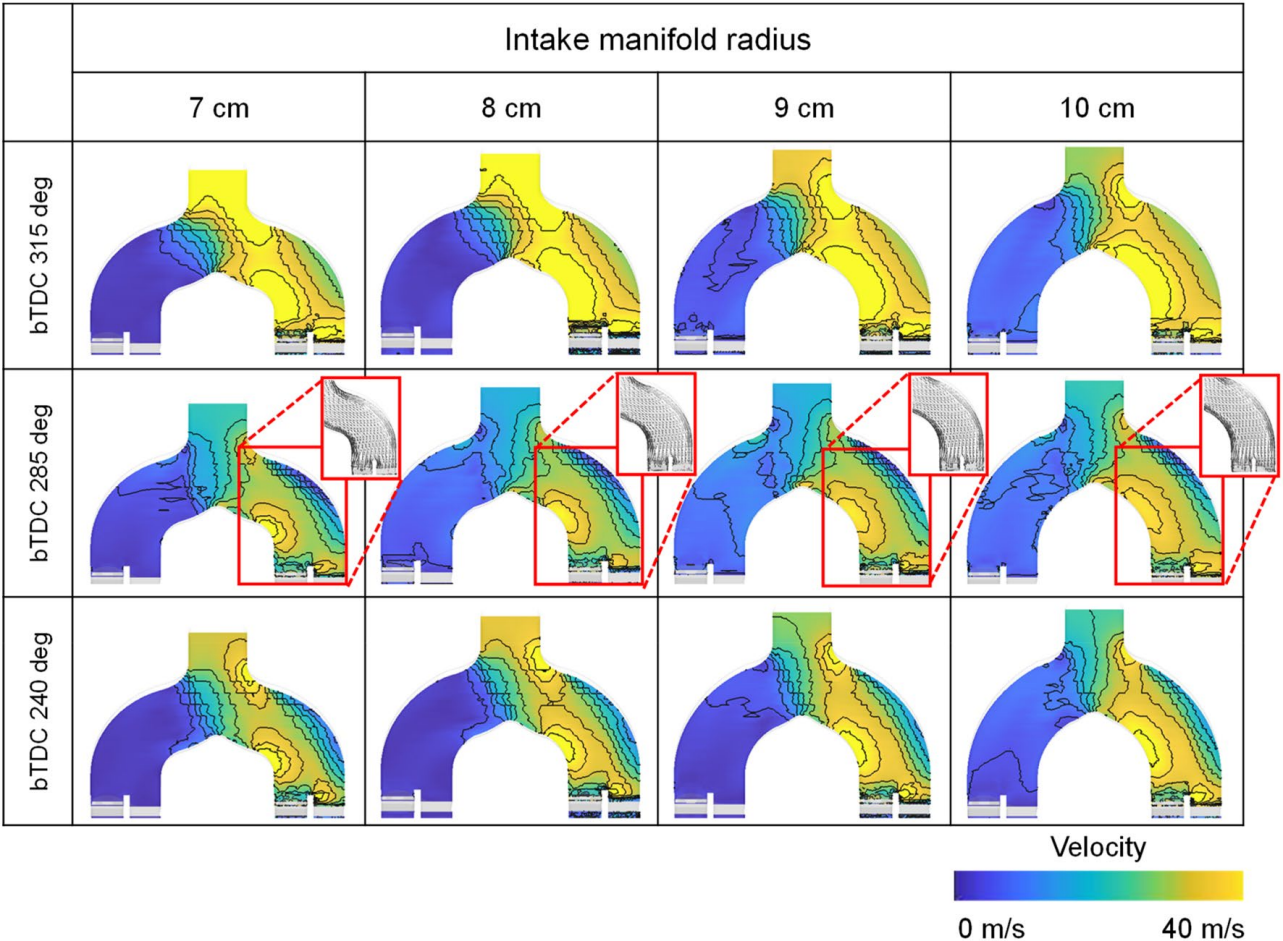
Velocity distribution at intake manifold during cylinder 1 intake process for different intake manifold curve radius at engine speed of 2000 rpm.

Figure 21 represents the velocity distribution at the intake manifold during the cylinder 1 intake process. As the intake manifold radius increased, the straightness of the flow inside the manifold is secured along the radius of the curved surface during rapid charging in early intake process. Therefore, it had a faster velocity distribution in the manifold due to a reduced momentum by colliding manifold surface.

**Figure 22:**
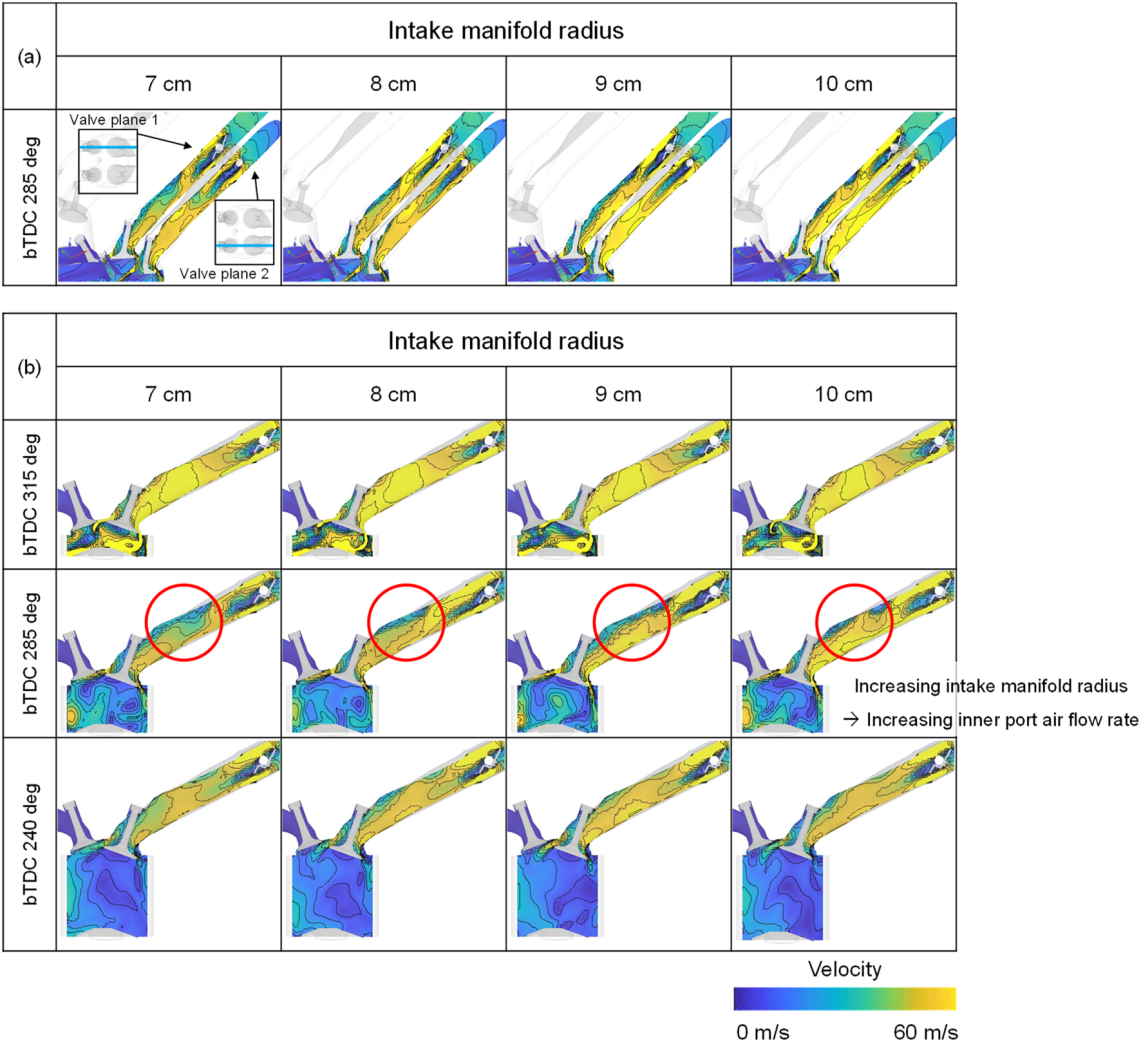
Velocity distribution in (**a**) valve planes and (**b**) valve plane 1 for different intake manifold curve radius at engine speed of 2000 rpm.

The difference in flow velocity in the intake port was caused by the difference in flow velocity and direction inside the intake manifold as shown in Fig. 22a. When the plane cut with respect to the inner port was referred to as valve plane 1 and the plane cut from the outer port was referred to as valve plane 2, a higher speed distribution could be confirmed at the inner port due to the characteristics of the straight intake manifold. Also, because of the influence of the flow inside the intake manifold, the flow inside the intake port was strong and evenly distributed as the intake manifold curve radius increased (Fig. 22b).

**Figure 23:**
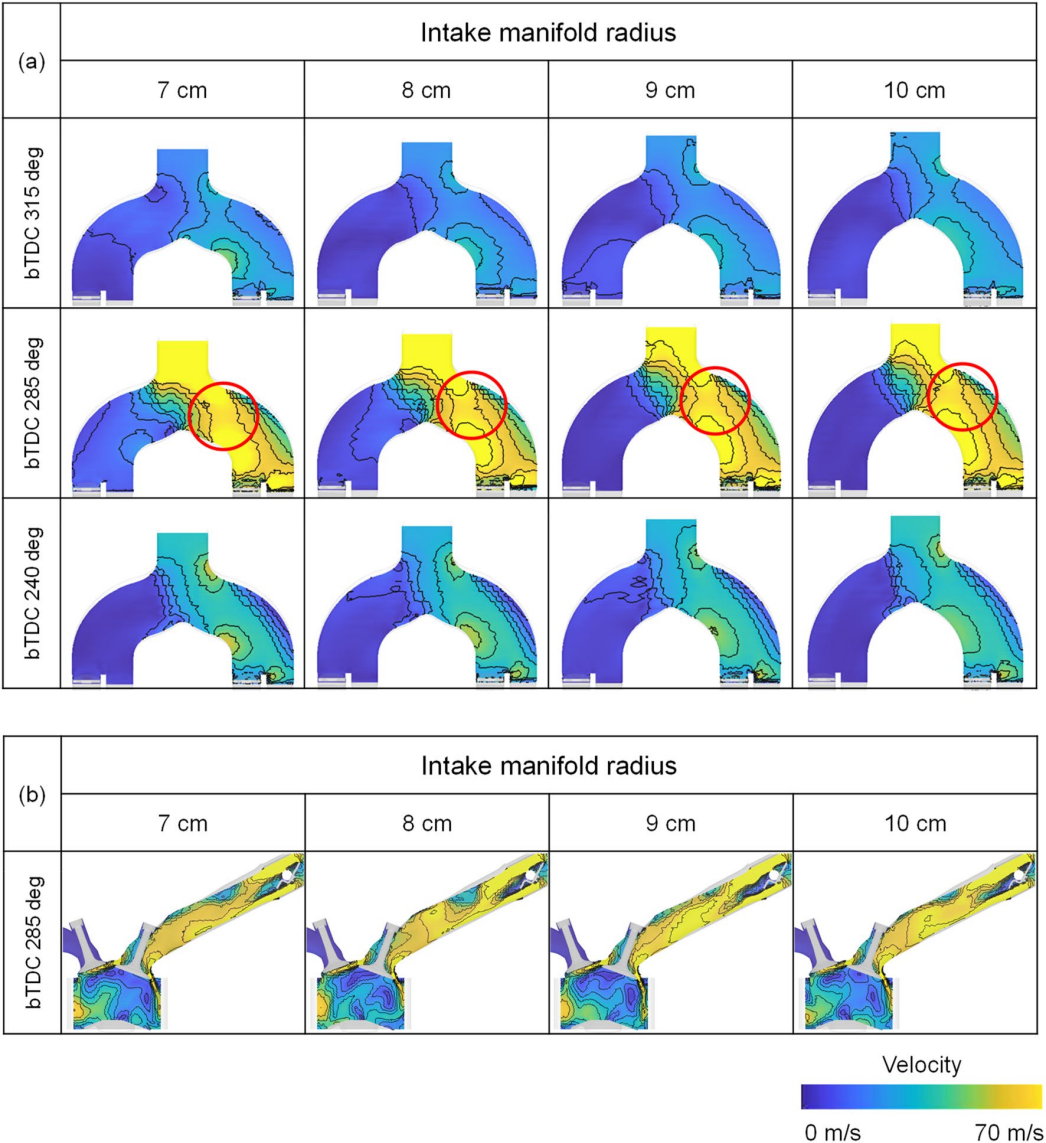
Velocity distribution in (**a**) intake manifold and (**b**) valve plane 1 for different intake manifold curve radius at engine speed of 3000 rpm.

However, with increasing engine speed, the rapid charging occurred, so that the distance between the intake manifold inlet and the port inlet also affected the intake process. At 3000 rpm, as the distance between the intake manifold inlet and the intake port increased, the response of the intake manifold deteriorated so that the velocity decreased in the region where flow was divided into each cylinder as shown in Fig. 23a. Therefore, when comprehensively considered including the effect with the intake manifold curve radius, the fastest air velocity within the intake port was shown at the intake manifold curve radius of 9 cm (Fig. 23b) in early intake process.

**Figure 24:**
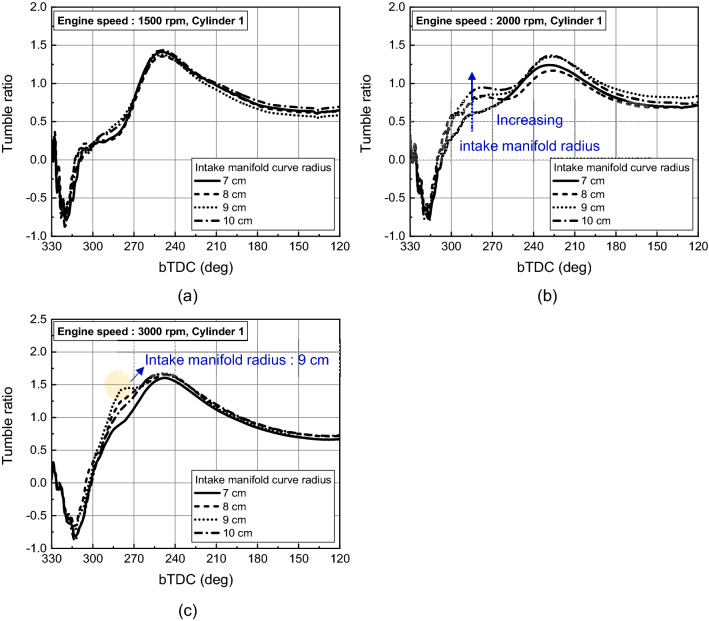
Tumble ratio for different intake manifold curve radius at engine speed of (**a**) 1500 rpm, (**b**) 2000 rpm, and (**c**) 3000 rpm.

The difference in flow velocity from the intake manifold and intake port influenced the in-cylinder intake velocity, which governed early tumble development. At engine speed of 1500 rpm and 2000 rpm, the early tumble ratio became stronger as the intake manifold curve radius increased. And at 2000 rpm, the trend was more pronounced with a tumble ratio increased 55% at intake manifold radius of 10 cm than of 7 cm at bTDC 280 deg as shown in Fig. 24a,b. On the other hand, at the engine speed of 3000 rpm, the distance between the intake manifold inlet and port also had an effect on the air charging process, so the early tumble was strongest with a tumble ratio value of nearly 1.5 at bTDC 280 deg at the intake manifold radius of 9 cm, while the tumble ratio value was less than 1.0 at intake manifold radius of 7 cm (Fig. 24c).

**Figure 25:**
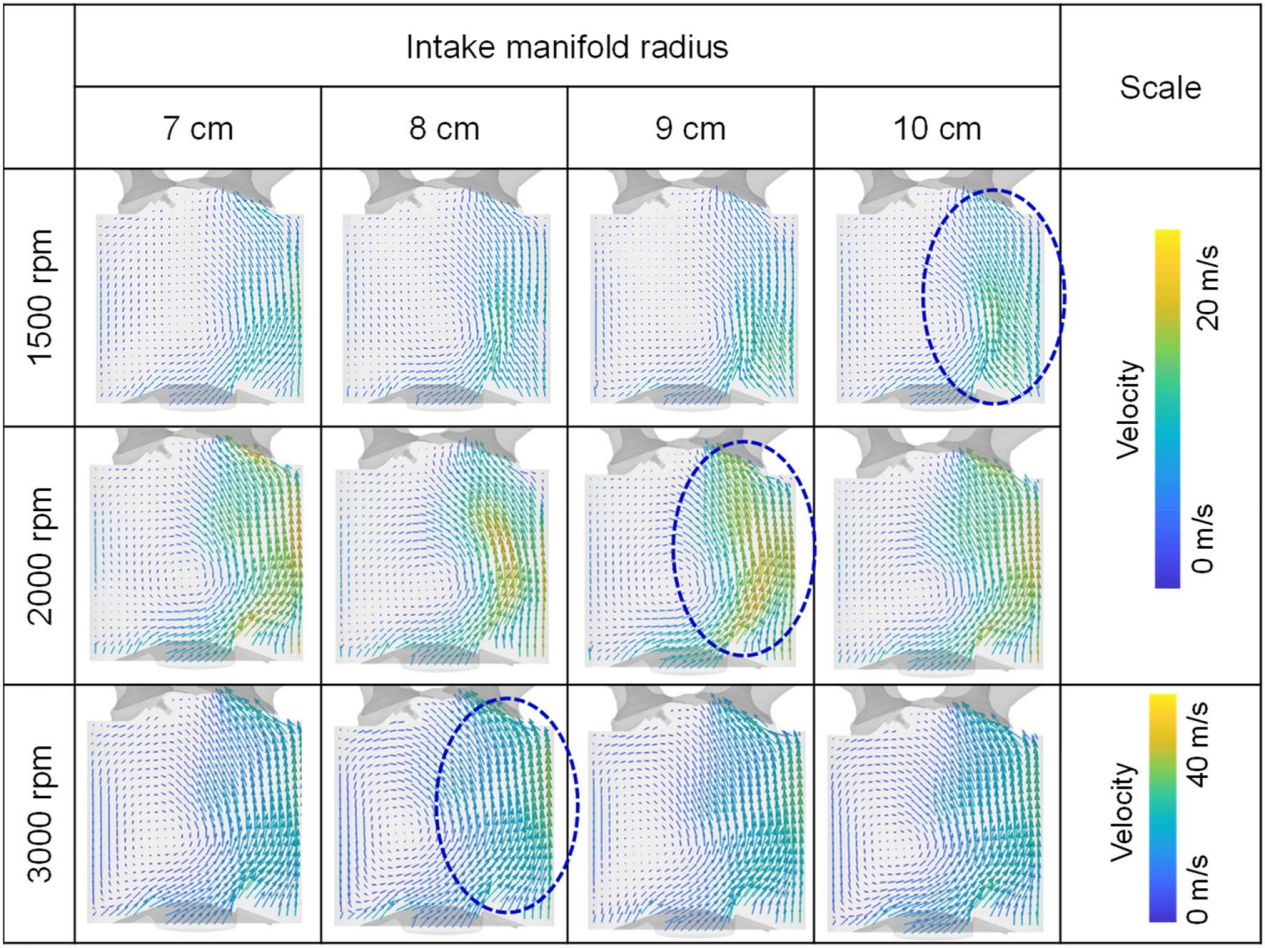
Velocity distribution for different intake manifold radius and engine speed at bTDC 120°.

The disturbance is generated by the early intake flow, and as the flow intensity increased, the more flow momentum loss by this disturbance increased. Therefore, the flow strength in the compression process depended on the early flow intensity and the resulting flow loss. And with increasing engine speed, the in-cylinder flow more affected by the distance from the intake manifold inlet and intake port than intake manifold radius. Therefore, at bTDC 120° which was during compression process, in the engine speed of 1500 rpm, the flow straightness was secured, and the strong in-cylinder velocity shown at intake manifold radius of 10 cm. However, as the engine speed increased, as the intake manifold inlet and the intake port distance got closer, i.e., the intake manifold radius decreased, the flow intensified as shown in Fig. 25.

**Figure 26:**
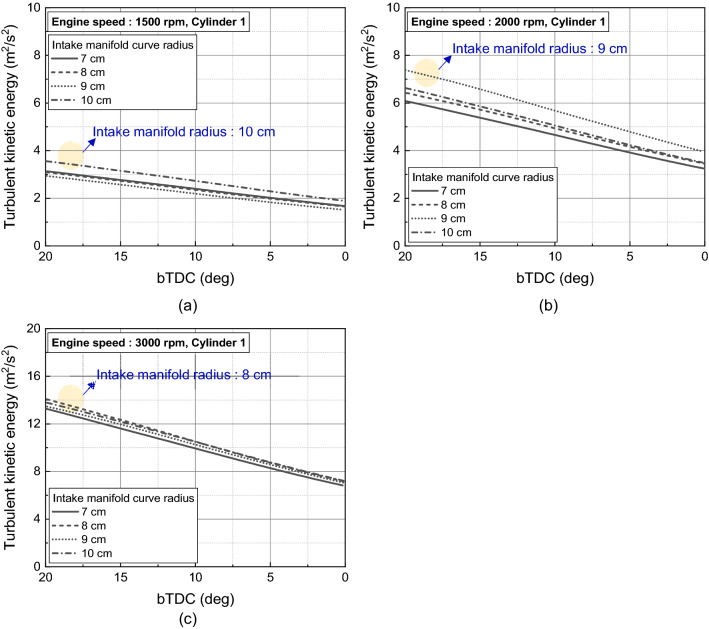
In-cylinder turbulent kinetic energy near TDC for different intake manifold radius at engine speed of (**a**) 1500 rpm, (**b**) 2000 rpm, and (**c**) 3000 rpm.

As the in-cylinder flow intensity during the compression process for various intake manifold radius was affected by the intake manifold radius itself and the distance between the intake manifold inlet and intake port, the turbulent kinetic energy near TDC reflected such a trend. As a result, the turbulent kinetic energy near TDC was highest in the intake manifold radius 10 cm, 9 cm, and 8 cm as the engine speed increased to 1500 rpm, 2000 rpm, and 3000 rpm (Fig. 26). Therefore, for the purpose of enhancing the in-cylinder flow and turbulence near spark plug timing, to apply the wide engine operating range, shortened distance between intake manifold inlet and port and increasing the manifold radius is required.

**Figure 27:**
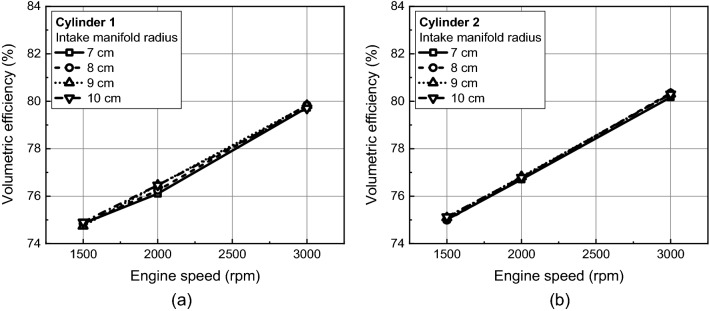
Volumetric efficiency for different intake manifold curve radius at (**a**) cylinder 1 and (**b**) cylinder 2.

Figure 27 represents the volumetric efficiency for different intake curve radius and cylinders. In cylinder 1 without a swirl valve in the inner port, the volumetric efficiency increased by 0.5% as the intake manifold cure radius increased from 7 to 10 cm at engine speed of 2000 rpm. However, there was no significant difference in the volumetric efficiency for different intake curve radius under other conditions.

## Conclusions

In this study, influence of intake manifold geometry on the cylinder-to-cylinder variation and in-cylinder flow characteristic were analyzed based on curved and straight intake manifold designs. Additionally, changes in the intake manifold radius were considered to enhance the in-cylinder flow for straight intake manifold. The main conclusions are as followed.

1. In curved intake manifold, the cylinder-to-cylinder variation was caused by the difference between the cylinder and intake manifold inlet. The cylinder-to-cylinder variation of volumetric efficiency was a maximum deviation of 1.7% and a minimum of 0.7% between cylinders. On the other hand, in the straight intake manifold, there was a maximum deviation of 0.6% and a minimum deviation of 0.1% between cylinders.
2. In curved intake manifold, the pressure fluctuation during the intake process of cylinder 1 lagged behind that of cylinder 2 so that the tumble variation between cylinders was larger than that of the straight intake manifold. And at the engine speed of 1500 rpm, the spark plug gap velocity variation at TDC between cylinder was reduced in straight manifold.
3. The straight manifold had the effect of strengthening the in-cylinder flow, so that the turbulent kinetic energy near TDC was increased to maximum 11.0% than curved manifold at engine speed of 2000 rpm.
4. When the intake manifold curve radius was increased in the straight manifold, the velocity inside the manifold was strengthened during early intake process, so that the flow rate in the intake port was increased at engine speed of 1500 rpm. However, with increasing engine speed, not only the intake manifold curve radius but also the distance between intake manifold inlet and the intake port affected to the intake process because of the rapid charging.
5. With increasing engine speed, the in-cylinder flow affected more by distance between intake manifold inlet and intake port than intake manifold radius. The turbulent kinetic energy near TDC was highest in the intake manifold radius 10 cm, 9 cm, and 8 cm as the engine speed increased to 1500 rpm, 2000 rpm, and 3000 rpm

## List of symbols

$k$: Turbulent kinetic energy
$\omega$: Angular speed of the flow around the center of mass in the tumble direction
${\omega }_{crank shaft}$: Angular speed of the crank shaft

## Abbreviations

GDI: Gasoline direct injection
CFD: Computational fluid dynamics
PIV: Particle image velocimetry
IVO: Intake valve open
IVC: Intake valve closed
EVO: Exhaust valve open
EVC: Exhaust valve closed
